# Supplementary material for: Prevalence, clinical presentation and factors associated with chronic lung disease among children and adolescents living with HIV in Kenya
Source: PLoS One. 2023 Aug 9;18(8):e0289756. doi: 10.1371/journal.pone.0289756 (PMC10411792; doi:10.1371/journal.pone.0289756)
Supplement: S1 Table — (PDF) [file pone.0289756.s002.pdf]

STROBE Statement: Prevalence, clinical presentation and factors associated with chronic lung disease (CLD) among children and adolescents with HIV in Kenya

(checklist of items that should be included in reports of observational studies)

	Item No.	Recommendation	Page No.	Relevant text from manuscript
**Title and abstract**	1	(*a*) Indicate the study's design with a commonly used term in the title or the abstract	3	Longitudinal cohort study as stated in the abstract on page 3
		(*b*) Provide in the abstract an informative and balanced summary of what was done and what was found	3	Provided in the Abstract on page 3.
**Introduction**				
Background/rationale	2	Explain the scientific background and rationale for the investigation being reported	4-5	Provided in the Introduction on pages 4-5.
Objectives	3	State specific objectives, including any prespecified hypotheses	5	Stated in the Introduction on page 5.
**Methods**				
Study design	4	Present key elements of study design early in the paper	5	Described in the Methods on page 5.
Setting	5	Describe the setting, locations, and relevant dates, including periods of recruitment, exposure, follow-up, and data collection	5	Described in the Methods on pages 5.
Participants	6	(*a*) *Cohort study*—Give the eligibility criteria, and the sources and methods of selection of participants. Describe methods of follow-up *Case-control study*—Give the eligibility criteria, and the sources and methods of case ascertainment and control selection. Give the rationale for the choice of cases and controls	5	Included in the Methods on page 5

		Cross-sectional study—Give the eligibility criteria, and the sources and methods of selection of participants		
		(*b*) *Cohort study*—For matched studies, give matching criteria and number of exposed and unexposed<br>*Case-control study*—For matched studies, give matching criteria and the number of controls per case		
Variables	7	Clearly define all outcomes, exposures, predictors, potential confounders, and effect modifiers. Give diagnostic criteria, if applicable	6-9	Included in the Methods on pages 6-9.
Data sources/ measurement	8*	For each variable of interest, give sources of data and details of methods of assessment (measurement). Describe comparability of assessment methods if there is more than one group	6-9	Included in the Methods on pages 6-9.
Bias	9	Describe any efforts to address potential sources of bias	9-10, 33-34	Described in the Methods data analysis section on pages 9-10, and in the Discussion on limitations on pages 33-34.
Study size	10	Explain how the study size was arrived at	9	Described in Methods section page 9.

Continued on next page

Quantitative variables	11	Explain how quantitative variables were handled in the analyses. If applicable, describe which groupings were chosen and why	8-9	Explained in clinical definitions section, and in the data analysis section of the Methods on page 8-9.
Statistical methods	12	(*a*) Describe all statistical methods, including those used to control for confounding	9-10	Indicated in the data analysis section of the Methods on pages 9-10
		(*b*) Describe any methods used to examine subgroups and interactions	9-10	Indicated in the data analysis section of the Methods on pages 9-10
		(*c*) Explain how missing data were addressed	9-10	Indicated in the data analysis section of the Methods on pages 9-10
		(*d*) *Cohort study*—If applicable, explain how loss to follow-up was addressed<br>*Case-control study*—If applicable, explain how matching of cases and controls was addressed<br>*Cross-sectional study*—If applicable, describe analytical methods taking account of sampling strategy	9	Indicated in Methods on page 9. This paper reports results the baseline findings of the cohort.
		(*e*) Describe any sensitivity analyses	10, 27	Indicated in data analysis section of methods on page 10, and in multivariable analysis results on page 27.
**Results**				
Participants	13*	(a) Report numbers of individuals at each stage of study—eg numbers potentially eligible, examined for eligibility, confirmed eligible, included in the study, completing follow-up, and analysed	5, 10	Indicated in study site description of methods on page 5, and in results on page 10. This manuscript reports baseline findings of all 320 participants at enrolment into the study. Follow-up results shall be reported in a separate manuscript.
		(b) Give reasons for non-participation at each stage	10-11	Indicated in Results page 10-11.
		(c) Consider use of a flow diagram	-	No flow diagram required, information adequately covered in opening sentences of Results section.
Descriptive data	14*	(a) Give characteristics of study participants (eg demographic, clinical, social) and information on exposures and potential confounders	10-14	Reported in Results pages 10-14, tables 1 – 2.
		(b) Indicate number of participants with missing data for each variable of interest	10-14	Reported in Results pages 10-14, tables 1 – 2.
		(c) *Cohort study*—Summarise follow-up time (eg, average and total amount)	-	No follow-up data reported in this manuscript which focuses on baseline results of the parent study.
Outcome data	15*	*Cohort study*—Report numbers of outcome events or summary measures over time	15-20	Baseline prevalence of chronic lung disease, and of specific clinical symptoms and signs of CLD (clinical

				presentation of CLD) are reported in this manuscript on pages 15-20, tables 3-4. Follow-up findings shall be analysed and reported in a separate paper.
		Case-control study—Report numbers in each exposure category, or summary measures of exposure	NA	
		Cross-sectional study—Report numbers of outcome events or summary measures	NA	
Main results	16	(*a*) Give unadjusted estimates and, if applicable, confounder-adjusted estimates and their precision (eg, 95% confidence interval). Make clear which confounders were adjusted for and why they were included	20-27	Indicated in Results section on factors associated with presence or absence of Chronic lung disease on pages 20-27, tables 5 – 7.
		(*b*) Report category boundaries when continuous variables were categorized	10-27	Indicated in results sections pages 10-27, tables 1 – 7 where appropriate.
		(*c*) If relevant, consider translating estimates of relative risk into absolute risk for a meaningful time period	NA	

Continued on next page

Other analyses	17	Report other analyses done—eg analyses of subgroups and interactions, and sensitivity analyses	27	Indicated in Results section on page 27, table 7.
**Discussion**				
Key results	18	Summarise key results with reference to study objectives	28, 38	Indicated in Discussion first paragraph page 28, and conclusions page 38.
Limitations	19	Discuss limitations of the study, taking into account sources of potential bias or imprecision. Discuss both direction and magnitude of any potential bias	33	Indicated in limitations section of Discussion page 33.
Interpretation	20	Give a cautious overall interpretation of results considering objectives, limitations, multiplicity of analyses, results from similar studies, and other relevant evidence	28-33	Provided in Discussion pages 28-33
Generalisability	21	Discuss the generalisability (external validity) of the study results	33-34	Indicated at end of discussion section, pages 33-34.
**Other information**				
Funding	22	Give the source of funding and the role of the funders for the present study and, if applicable, for the original study on which the present article is based	-	Kenyatta National Hospital, Grant no. KNH/R&P/23 1/3/11. Funding institution is the hospital in which the study participants receive routine care.

*Give information separately for cases and controls in case-control studies and, if applicable, for exposed and unexposed groups in cohort and cross-sectional studies.

Note: An Explanation and Elaboration article discusses each checklist item and gives methodological background and published examples of transparent reporting. The STROBE checklist is best used in conjunction with this article (freely available on the Web sites of PLoS Medicine at http://www.plosmedicine.org/, Annals of Internal Medicine at http://www.annals.org/, and Epidemiology at http://www.epidem.com/). Information on the STROBE Initiative is available at www.strobe-statement.org.
