# Supplementary material for: Prevalence, clinical presentation and factors associated with chronic lung disease among children and adolescents living with HIV in Kenya
Source: PLoS One. 2023 Aug 9;18(8):e0289756. doi: 10.1371/journal.pone.0289756 (PMC10411792; doi:10.1371/journal.pone.0289756)
Supplement: Attachment — Submitted filename: Response to Reviewers.pdf [file pone.0289756.s003.pdf]

UNIVERSITY OF NAIROBI
FACULTY OF HEALTH SCIENCES
SCHOOL OF MEDICINE
DEPARTMENT OF PAEDIATRICS AND CHILD HEALTH

Telephone: +(254) 20 4915046
Email : dept-paediatrics@uonbi.ac.ke

Kenyatta National Hospital
P.O. Box 19676
Nairobi, Kenya. 00202

To:
The Academic Editor,
PLoS One

20th July 2023

Dear Dr Kose,

RE: Rebuttal Letter & Response to Reviewers: Prevalence, clinical presentation and factors associated with chronic lung disease among children and adolescents living with HIV in Kenya (PONE-D-23-16528)

Thank you very much for reviewing our above-mentioned scientific research manuscript and for giving us an opportunity to address the reviewers' comments. The comments were helpful and valuable to improve and refine the manuscript. We have revised the manuscript accordingly and submit both track changes and clean versions of the revised manuscript. We have made a point-by-point response to the comments of both the editor and the reviewers, each response is inserted below the specific comment. All the required documents have been submitted using the online system.

We do hope that we have responded adequately to the comments of the reviewers, and that we have addressed all the requirements of the editors and journal. Once again, we thank the Editors of PLOS One and the reviewers for your timely review of this scientific research manuscript on the prevalence, clinical presentation and factors associated with chronic lung disease among children and adolescents living with HIV in Kenya.

Our detailed point by point responses are provided below:

Academic Editor Requirements:

Please include the following items when submitting your revised manuscript:

- A rebuttal letter that responds to each point raised by the academic editor and reviewer(s). You should upload this letter as a separate file labeled 'Response to Reviewers'. DONE
- A marked-up copy of your manuscript that highlights changes made to the original version. You should upload this as a separate file labeled 'Revised Manuscript with Track Changes'. DONE
- An unmarked version of your revised paper without tracked changes. You should upload this as a separate file labeled 'Manuscript'. DONE

Journal Requirements:

When submitting your revision, we need you to address these additional requirements.

1. Please ensure that your manuscript meets PLOS ONE's style requirements, including those for file naming. The PLOS ONE style templates can be found at https://journals.plos.org/plosone/s/file?id=wjVg/PLOSOne_formatting_sample_main_body.pdf and https://journals.plos.org/plosone/s/file?id=ba62/PLOSOne_formatting_sample_title_authors_affiliations.pdf

Response: We have formatted our manuscript to conform to PLOS ONE's style requirements.

2. Please include a complete copy of PLOS' questionnaire on inclusivity in global research in your revised manuscript. Our policy for research in this area aims to improve transparency in the reporting of research performed outside of researchers' own country or community. The policy applies to researchers who have travelled to a different country to conduct research, research with Indigenous populations or their lands, and research on cultural artefacts. The questionnaire can also be requested at the journal's discretion for any other submissions, even if these conditions are not met. Please find more information on the policy and a link to download a blank copy of the questionnaire here: https://journals.plos.org/plosone/s/best-practices-in-research-reporting. Please upload a completed version of your questionnaire as Supporting Information when you resubmit your manuscript.

Response: The lead researchers and co-investigators are Kenyan citizens and conducted research within our own country Kenya. The policy therefore may not really apply to us, nevertheless I have filled this form as requested.

3. Please include your full ethics statement in the ‘Methods’ section of your manuscript file. In your statement, please include the full name of the IRB or ethics committee who approved or waived your study, as well as whether or not you obtained informed written or verbal consent. If consent was waived for your study, please include this information in your statement as well.

Response: We have included this information in the Methods section of the manuscript.

4. Please review your reference list to ensure that it is complete and correct. If you have cited papers that have been retracted, please include the rationale for doing so in the manuscript text, or remove these references and replace them with relevant current references. Any changes to the reference list should be mentioned in the rebuttal letter that accompanies your revised manuscript. If you need to cite a retracted article, indicate the article’s retracted status in the References list and also include a citation and full reference for the retraction notice.

Response: We have done so. One new reference was added in methods section (ref 22). One reference citation which we had inadvertently duplicated in the ref list (was cited twice as ref 11 and ref 39) this was corrected. Total number of references remains 41.

New reference no. 22:

ATS committee on proficiency standards for clinical pulmonary function laboratories. ATS statement: guidelines for the six-minute walk test. Am J Respir Crit Care Med. 2002 Jul 1;166(1):111-7. doi: 10.1164/ajrccm.166.1.at1102. Erratum in: Am J Respir Crit Care Med. 2016 May 15;193(10):1185. PMID: 12091180.

Duplicated reference (pointed out by the second reviewer) now cited once as ref no. 11:

Verwey C, Gray DM, Dangor Z, Ferrand RA, Ayuk AC, Marangu D, et al. Bronchiectasis in African children: challenges and barriers to care. Front Pediatr. 2022 Jul 25; 10:954608. doi: 10.3389/fped.2022.954608. PMID: 35958169; PMCID: PMC9357921

Responses to Reviewer Comments

Reviewer 1

Abstract
Line 43: not clear what is the difference between exertional breathless and exercise limitation line 45.

Response: Exertional breathlessness – this was the response to the question "do you get short of breath when you climb stairs or when walking uphill?". This question was posed to the child/adolescent during the interview.

Exercise limitation – this refers to limitation in physical activity. This was assessed using a standardized exercise test – the six-minute walk test commonly used to assess if an individual has limitation in physical activity, or exercise limitation (Ref 22). A participant walks as fast as they can on flat ground for six minutes, and the total distance walked is recorded. The distance achieved is then compared against a benchmark of the expected distance that a healthy child of similar height, weight and age should walk, and reported as a percentage of predicted distance (ref 25). Details on the methods and classification of exercise limitation are provided in methods section of the manuscript (manuscript with tracked changes: p6, lines 135-137, p8 lines 170-172).

Methods
Can the authors reference their definition of enhanced chronic lung disease (CLD)? Is their definition of enhanced CLD in literature?

Response: There are no standardized definitions of chronic lung disease for HIV infected children and adolescents, and researchers have used varying definitions tailored to their specific studies and clinical settings, these studies are mentioned in the introduction section of the manuscript (line 89, refs 10-11, 15-21). They are further mentioned in the discussion section (p31, lines 548 – 554, refs 17, 18).

We examined the various definitions in published literature in CAHIV on this subject, when considering which symptoms and signs to use in the CLD definition in our study. The commonest symptoms suggestive of CLD used across studies are chronic cough, and breathlessness. Common physical examination signs suggestive of CLD used across studies include finger clubbing, and abnormal oxygen saturation were also popular. Some studies subjected participants to exercise but the outcome parameters used were exercise induced breathlessness, and hypoxia/desaturation, and the exercise tests used differed from ours (p20 – 30, lines 502-511, 519 – 527, 538-542, refs 16-18, 28-32). We found one study by Githinji et al on CAHIV that conducted the six-minute walk test and measured walk distance as an outcome of interest, however they did not use this parameter as part of diagnosis for CLD (p30,line 536-538, ref 36). They conducted extensive lung function testing as their main diagnostic tool for diagnosis of CLD.

Results
Table 1: what is the rationale of grouping children into <7 years, <7-9 years? Why not under 5 years category and 5-9 years old, for instance?

Response: We noted from review of literature that previous researchers had excluded children below 7 years – so our study is the first to provide insight on this problem among children <7 years. For this reason we thought it useful to display the number of children of age-group <7 yr that we enrolled in this study. We have added a footer to table 1 stating: "Additional detail on children <10 yrs: 14 (4.4%) were age <5 yr; 66 (20.6%) were age 5-9 yr." (p12, line 261).

Line 247: what does the term "chest problem" refer to?

Response: The participants were asked "In the last 12 months have you had a chest problem, outside of a common cold?"

Further details of the chest problem were also asked – specifically we asked if they had experienced congested chest, tight chest, or chest pain in the preceding 12 months.

Table 4: CLD and CLD enhanced-are not those with breathlessness double counted with those with exercise limitation and vice versa?

Response: Thank you for this thoughtful query. We also considered this when examining our findings. In this study population some children experienced neither symptom, some experienced one and not the other, whereas some experienced both breathlessness and exercise limitation. The latter group would then be identified with two symptoms suggestive of CLD, but those with only one of the two criteria would not be identified as CLD based on these two specific clinical features. It is unclear why some breathless children achieved normal walk distance and others did not, and vice versa. We do note that the enhanced definition appears to capture additional subjects who were not identified using the basic CLD approach.

There is indeed need for further research to better elucidate the pathophysiology leading to breathlessness and to exercise limitation, and the interaction between the two, as well as other clinical symptoms and signs suggestive of chronic lung pathology.

Line 348 "took INH prophylaxis later" Please rephrase this statement to denote late initiation of INH prophylaxis

Response: This has been rephrased. (p21, Line 420)

Line 353 "pneumonia" does this term perhaps refer to non-TB lower respiratory tract infection?

Response: The participants were asked if "Have you ever had pneumonia or chest infection other than TB". In general any acute chest infection is described as "pneumonia" by non-medical persons in our setting, and is not distinguished from bronchiolitis or bronchitis.

Can the term enhanced CLD be referenced from published literature?

Response: The term is "enhanced definition of CLD" rather than "enhanced CLD". This is a term specific to this study. We have given more detailed response to this in the earlier comment raised by the reviewer, please see earlier section.

Discussion

Line 399: exertional breathless vs exercise limitation. Please see my comment above. Could the reduced distance walked during 6MWT in Table 4 be due to low oxygen saturation or breathlessness and hence this sub-group is already counted in the above?

Response: Thank you for your thoughtful analysis of the various scenarios. We concur that exercise induced symptoms may then lead to a child slowing down and this in turn lead to reduced distance walked.

We tried to be conservative in the definition of exercise limitation and used a cut-off of "achieved <70% of predicted distance" or moderate limitation as opposed to <80% of predicted (includes those with mild exercise limitation) in order to minimize over-calling exercise limitation (p8, line 186 – 191).

In this study population all the 17 participants who experienced oxygen desaturation of 3% or more were identified in the basic CLD group, in other words all 17 had at least one additional symptom/sign (cough, breathlessness, tachypnoea or finger clubbing) regardless of distance walked.

Some children in our study reported being short of breath during the walk, but it did not limit their walk distance, and this group of children the enhanced CLD approach did not re-categorize them as CLD if they had none of the other four symptoms/signs. It is possible that children who got breathless but had no other respiratory symptoms or signs (suggestive of healthy respiratory system) and had normal walk distance were simply a bit physically unfit. Such a child would not be categorized as having CLD either with the basic definition or the enhanced definition.

The□uestionn that you raise points to a need for further research that would provide insight into the pathologic changes and pathophysiology of CLD, and the relative importance of each specific respiratory symptom and sign in diagnosing CLD in these vulnerable CAHIV. We attempted to articulate some of these issues in the discussion of these findings.

Line 433 “chest problems” does this term refer to pain, infections, tight chest, etc?

Response: The participants were asked “In the last 12 months have you had a chest problem, outside of a common cold?”

They were also asked what type of chest problem they had experienced – specifically we asked if they had experienced congested chest, tight chest, or chest pain in the preceding 12 months.

Line 497 the authors state that stunting and CLD implication of management is uncertain. Is this true, considering stunting denotes chronic malnutrition and nutrition impacts lung growth; height for instance impacts lung function.

Response: Thank you for your thoughtful queries to this statement. Given that the height and CLD assessment were done at the same point in time, it is not possible to know which occurred before the other. We do concur with your observation that stunting may denote chronic malnutrition and nutrition impacts lung growth. However we were also cognissant of the fact that in these CAHIV causes of stunting may be multifactorial, due to chronic inadequate nutrition, or due to other HIV-related morbidity, and in CLD may be due to chronic sub-optimal oxygenation, or recurrent lung infections due to damaged lungs. For this reason we were cautious in our interpretation of this finding and its implications for management.

References

Refs 11 and 39 are the same, Verwey, et al.

Response: Thank you for pointing this out, we have deleted the duplicate ref 39, and re-aligned the references as appropriate.

Reviewer 2

1. This is an interesting study addressing a topic with clear scientific value. Due to the period of the study (covid-19), with the exception of the standardized six-minute walk test (6MWT) being carried out outdoors for safety precautions, no other implications of covid-19 are mentioned in the study results. Evaluate and discuss implications of covid-19 on study results, if applicable.

Response: Thank you for this comment, it is an important factor to consider. We have added a statement on effect of covid-19 pandemic at the beginning of the results (p10, lines 232 - 234). The statement reads as follows:

"Due to the covid-19 pandemic active enrolment was stopped on 18th March 2020 after enrolling the first 14 participants, and restarted eight months later on 25th November 2020 with covid-19 precautions in place for participants and staff".

The major implication for the study is that enrolment took longer than initially anticipated, however we were still able to achieve the targeted sample size required for the study, and participants were able to complete the interview, physical examination and exercise testing since the latter was done outdoors. By the time we restarted the study parents were generally continuing with normal work and other activities, children were on school vacation and interacting freely within their neighborhoods. Very few study children (only two that we are aware of) were diagnosed with covid-19 during the study period, however access to testing was low due to the high cost of the test, and the low socio-economic status of our study population. The two children had mild covid-19 symptoms and recovered fully.

We see no additional significant implication of covid-19 on the study results.

2. There is need to provide a study profile including n of screened participants, n of excluded and n of participants included in the different evaluations for a understanding of the reader.

Response: Thank you for bringing this to our attention.

Details of screening, exclusion, enrolment and completion of clinical evaluations have been added in the first paragraph of the results (p10, line 229 – 232, 234 - 244).

Briefly we screened 326, two were ineligible as they were from a children's home, and four potentially eligible children declined to participate due to non-interest. Of the 320 enrolled, all completed the interview, all underwent physical examination and pulse oximetry assessment, and 312 completed the six-minute walk exercise test.

There was very high acceptance by study participants, perhaps they felt it was beneficial to know the status of their (their child's) lungs. The interviews and

procedures were carried out largely by clinicians and nurses who had cared for the children and adolescents for many years, so they had good rapport with them.

3. Actors should mention in the article the definition of outdoor pollution exposure definition.

Response: We have added details on definition of outdoor pollution exposure in the methods section of the manuscript. (p7, line 157 – 160)

4. Although the article collected information on the level of education, this seems to me to be decisive for older adolescents. In younger children, the child's health is mostly determined by the caregiver's education and not the child's. A limitation of the study is the fact that there is no information on the primary caregiver's education and also no information on who the questionnaire was administered to.

Response: Thank you for this insightful comment, we concur it is an important characteristic to evaluate. We had omitted this data due to the size of the manuscript. We have now added the caregiver characteristics in table 1 (page 12). Caregiver education data available for 282 (88%) of 320 participants. We have added a summary of this information in the narrative. (p12-13, lines 264 – 269). Association between caregiver education and CLD is reported on Table 5 (p20-21, lines 401 – 407. Table 5 p23). Caregivers with fewer years of education (1-8 yrs) had higher odds of their child having CLD thank those with >12 years education (crude OR 2.46, 95% CI 1.10 – 5.50, p=0.028).

Due to missing data on >10% of participants, per the a-priori multivariable model statistical plan this was not added to the adjusted analysis. However caregiver education is associated with socio-economic status, which was represented in the final multivariable models by the variable number of rooms in the home, which was an independent predictor of CLD in this study population.

====================================

Comments to the Author

1. Is the manuscript technically sound, and do the data support the conclusions?

The manuscript must describe a technically sound piece of scientific research with data that supports the conclusions. Experiments must have been conducted rigorously, with appropriate controls, replication, and sample sizes. The conclusions must be drawn appropriately based on the data presented.

Reviewer #1: Yes

Reviewer #2: Yes

---

2. Has the statistical analysis been performed appropriately and rigorously?

Reviewer #1: Yes

Reviewer #2: Yes

---

3. Have the authors made all data underlying the findings in their manuscript fully available?

The PLOS Data policy requires authors to make all data underlying the findings described in their manuscript fully available without restriction, with rare exception (please refer to the Data Availability Statement in the manuscript PDF file). The data should be provided as part of the manuscript or its supporting information, or deposited to a public repository. For example, in addition to summary statistics, the data points behind means, medians and variance measures should be available. If there are restrictions on publicly sharing data—e.g. participant privacy or use of data from a third party—those must be specified.

Reviewer #1: Yes

Reviewer #2: Yes

---

4. Is the manuscript presented in an intelligible fashion and written in standard English?

PLOS ONE does not copyedit accepted manuscripts, so the language in submitted articles must be clear, correct, and unambiguous. Any typographical or grammatical errors should be corrected at revision, so please note any specific errors here.

Reviewer #1: Yes

Reviewer #2: Yes

Concluding Remarks.

The authors of the manuscript titled “Prevalence, clinical presentation and factors associated with chronic lung disease among children and adolescents living with HIV in Kenya (PONE-D-23-16528)” appreciate the careful review and comments by the two reviewers, and by the academic editor. We do hope that we have responded adequately to the comments of the reviewers, and that we have addressed all the requirements of the editors and journal.

We look forward to hearing from you.

Yours Sincerely,

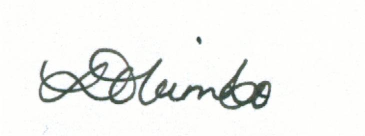

Elizabeth Maleche-Obimbo
Professor of Paediatrics & Respiratory Medicine
Epidemiologist and Paediatric Pulmonologist
Department of Paediatrics & Child Health
Faculty of Medicine
University of Nairobi.
Email: Elizabeth.obimbo@uonbi.ac.ke
